# Surgical Strategies in Total Colonic Aganglionosis: Primary Pullthrough-Pathway of Care

**DOI:** 10.3390/children11080911

**Published:** 2024-07-28

**Authors:** Stefanie Märzheuser, Felix Schulze, Judith Lindert

**Affiliations:** Department of Paediatric Surgery, University Hospital Rostock, Ernst-Heydemann Str 8, 18057 Rostock, Germany; stefanie.maerzheuser@med.uni-rostock.de (S.M.); felix.schulze@med.uni-rostock.de (F.S.)

**Keywords:** total colonic aganglionosis, surgical preparation, colonic pullthrough, ileoanal anastomosis, bowel management, Hirschsprung Disease

## Abstract

**Background:** Total colonic aganglionosis, as a rare variant of Hirschsprung Disease, still poses challenges to surgeons in terms of diagnosis and management. The optimal preparation for pullthrough is crucial for reconstructive surgery. This study aims to explore our surgical pathway for children with total colonic aganglionosis (TCA) and to describe the prehabilitation necessary to prepare for successful reconstructive pullthrough surgery. **Methods:** A prospective review of children with TCA receiving an abdominal surgical intervention between 1/22 and 4/24. The cohort included children receiving mapping +/− primary ileoanal pullthrough. An analysis of preoperative, perioperative, and postoperative data, and a short-term follow-up were performed. **Results:** A total of 18 children with TCA and no prior pullthrough received an abdominal intervention during the 29-month study period, and 5/18 (27.8%) were female. The children had a median of 4 (range 2–7) prior external surgeries; all had a stoma; 6 (33%) children received parental nutrition; 12 children underwent a mapping of the ganglia distribution and bowel length at a median age of 11 months (range 3–54), and in 10 of them, we relocated the stoma. There was a mean involvement of 15 (5–93) cm small bowel aganglionosis, with the remaining mean ganglionic small bowel having a length of 178 cm (110–254). A total of 11 children underwent straight primary ileoanal pullthrough of the stoma site at a median age of 16.7 months (10–133). **Conclusions:** The timely diagnosis of TCA still challenges the care team, and most children have a rough journey involving several surgeries until their diagnosis is established. The ensure bowel function with an adequate working stoma is the key to enabling enteral nutrition and growth, which are the baseline requirements to undertake a successful pullthrough procedure and restore continuity. Careful perioperative bowel management and parents’ active involvement supports children with Hirschsprung Disease achieving the best possible quality of life.

## 1. Introduction

Total colonic aganglionosis (TCA) affects 2–10% of all children with Hirschsprung Disease [1,2,3,4]. There is uncertainty as to whether it is a phenotype of Hirschsprung Disease or a separate entity. The extent of aganglionosis can be more proximal, and there seems to be agreement on the definition of total colonic aganglionosis with aganglionosis of the entire colon and up to 40–50 cm of the ileum proximal to the ileocecal valve (ICV) [5,6]. More extensive forms extending further into the colon are considered a separate condition [1].

Total colonic aganglionosis continues to challenge the medical team in terms of the initial diagnostic suspicion and subsequent appropriate management [7,8,9]. Several surgical techniques are used, including straight ileoanal pullthrough, Duhamel, and pouch creation with or without a protective stoma [7,10,11]. 

Previously, experts suggested that the ideal time for pullthrough was around the age of 3, when the child is toilet-trained for urine [9]. In addition, a permanent stoma has been advocated by several groups as the definitive treatment option [2,9]. However, during the recent scientific discussion, experts have shifted to recommending pullthrough surgery earlier, and experienced surgeons suggest focusing more on the clinical status and development of the child [12]. It is well known that the immediate postoperative period is often challenged by high stool frequency and perianal skin excoriation [8,12]. 

Due to its rare nature, most series follow a small number of patients, often over a long period of time with changing medical approaches and attitudes. In this manuscript, we analyze our recent cohort and describe preoperative preparation, operative characteristics, and immediate postoperative management in a cross-sectional study. The combination of a retrospective chart review and prospective follow-up allows a comparative assessment of short- and long-term outcomes. We analyze the preparation for primary pullthrough, appropriate bowel management, nutritional status, and identification of the right time for reconstructive surgery in our perioperative strategy.

## 2. Materials and Methods

**Figure 1:**
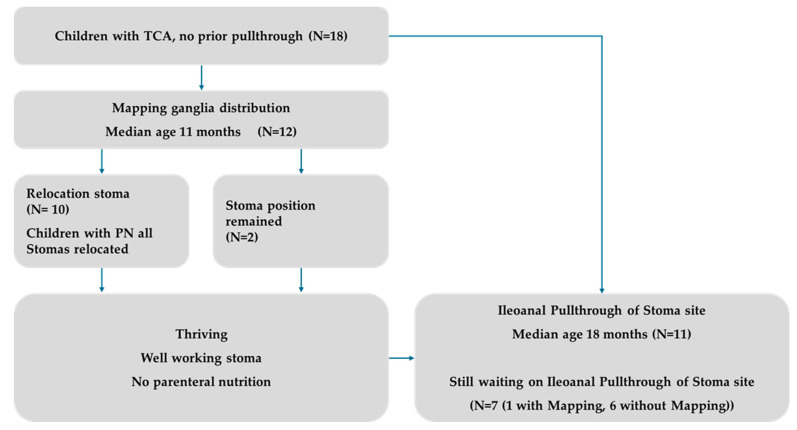
Workflow of the study population. Patients who had sufficient evidence and preliminary examinations in external hospitals did not require parenteral nutrition and had no complaints with the stoma received the primary draught directly. A total of 7 patients are currently still waiting for their surgery.

This was a cross-sectional study with a retrospective analysis of prospectively collected data of children with TCA operated at our institution during the 29 months from January 2022 to May 2024. We analyzed the clinical records of all patients with TCA who underwent an abdominal surgical intervention during the study period (N = 27), which included undergoing a mapping +/− ileoanal pullthrough (see Figure 1). We excluded revisional pullthrough operations during that time (N = 7). A total of 18 patients with no prior pullthrough were included in this analysis. Clinical examination and review were consistently performed by the same team for all patients.

Data on sex, associated malformations or syndromes, initial symptoms, age at referral, surgical history, nutritional status at presentation and surgery, age and anthropometric measurements at surgery, type of surgery, length of resected aganglionic bowel segment, and postoperative recovery (return of enteral intake, bowel function) were prospectively entered into our institutional database from the medical records. Follow-up data on bowel function, perianal skin condition, and episodes of enterocolitis were collected from the medical records. Our institutional holistic preparation and follow-up were applied to all patients and are illustrated in Table 1 and Table 2.

Growth, stool frequency, bowel management, episodes of enterocolitis, and perianal skin condition were assessed and monitored. 

**Definition and Diagnosis of Total Colonic Aganglionosis**: TCA is defined by the absence of ganglia in the entire colon with and without involvement of the small intestine. The diagnosis is established with a rectal biopsy and several leveling biopsies from the colon. If biopsies are from external institutions, we review the original pathology report, and if in doubt, we repeat biopsies or the leveling mapping process. If no prior mapping in our institution is needed, at least one colonic biopsy is taken as a frozen section and immediate reporting before the removal of the entire colon.

Definition of **toilet-trained for urine**: If the patient was dry and in normal underwear during the day, we considered the patient toilet-trained for urine [12]. 

Definition of **perianal skin condition**: A: skin intact, no erythema; B: skin intact, slight erythema; C: some small skin breakdown, erythema; D: skin breakdown of larger areas, erythema, moist lesion [13].

For the standardized follow-up (see Table 3), we see the patients one week after discharge to assess the early postoperative situation, to catch up on questions, and to fully prepare the families for home. We then systematically see all patients at 6 weeks and 3 months after ileoanal pullthrough. During the 3-month post-pullthrough visit, we assess the anastomosis once with a digital examination. No anal dilatations are performed. Follow-up visits are scheduled every 6 months for the first 2–3 years and then at intervals according to clinical need. We offer telemedicine support in-between if needed. Follow-up visits include a routine ultrasound of the bowel [14], standardized assessment of bowel function, and ongoing bowel management, nutritional status, perianal skin conditions, and overall child development. Depending on clinical status and distance to us, we offer telemedicine follow-up visits when appropriate.

Data are presented as median and range or mean and standard deviation, frequencies or numbers. For dichotomous data, the nonparametric Fisher exact test was used. For continuous data, the Mann–Whitney U test was used. Statistical analysis was performed with SPSS^®^ software version 26.0 (IBM, Armonk, NY, USA). *P* values of 0.05 were considered statistically significant. 

The study was approved by the Ethics Committee of the University Hospital Rostock (protocol code 2022-0187 on 30 November 2022).

## 3. Results

A total of 18 children with TCA without prior pullthrough received abdominal surgery during the study period of the 29 months between January 2022 and May 2024. We describe the pathway of care and the perioperative strategy for mapping the preparation for ileoanal pullthrough and the surgical strategy in primary pullthrough. Patient information: 5/18 female; children underwent mapping he children had a median of 4 (range 2–7) prior external surgeries; all children had a stoma when presenting to us. 6 (33%) children received parental nutrition; 12 children underwent a mapping of the ganglia distribution with us at a median age of 11 months (range 3–54), and in 10 of them, we relocated the stoma. There was a mean involvement of 15 (5–93) cm small bowel aganglionosis, with the remaining mean ganglionic small bowel having a length of 178 cm (110–254). A total of 11 children subsequently underwent straight primary ileoanal pullthrough of the stoma site at a median age of 16.7 months (10–133).

### 3.1. Patient Characteristics at Presentation to Us

When self-referred at a median age of 10 (2–38) months, these children had a median of 4 (2–7) prior external abdominal surgeries. We excluded any procedures for other reasons such as central lines or unrelated surgical problems from this count. All 18 children had a stoma at presentation; 10 had problems with their stoma: 7/10 obstruction, 2/10 prolapse, 2/10 Stenosis. A total of 6/18 (33%) children were receiving parental nutrition. Characteristics of the total group and children with and without previous pullthrough are shown in Table 4.

### 3.2. Patient Characteristics at Mapping and Findings

During the intestinal mapping procedure, we relocated the stoma in 65% (N = 11) of the children, of whom 6 were on parenteral nutrition (PN). The average aganglionic small bowel length involved was 25 cm. The average length of ganglionic small bowel was 201 cm (110–340). The mapping findings are summarized in Table 5.

### 3.3. Patient Characteristics at the TIME of Pullthrough Surgery

A total of 11 children underwent primary straight ileoanal pullthrough (median age 18 months (10–133) during the observation period and no stoma was left in place. Seven (70%) of them had completed prehabilitation after stoma relocation during mapping within the study period. See patient characteristics at pullthrough surgery in Table 6.

**Figure 2:**
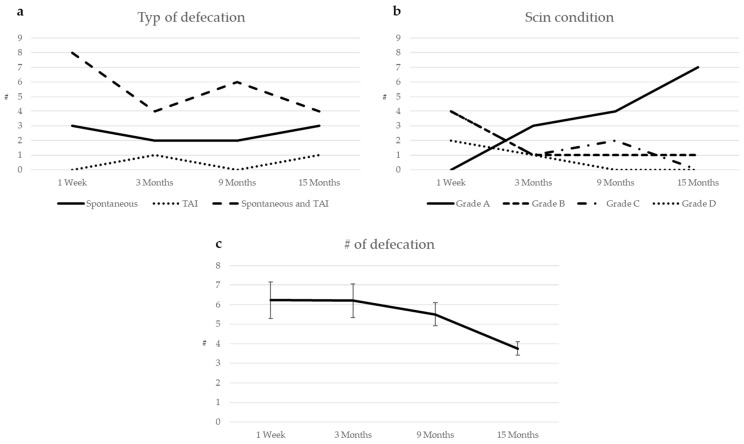
Follow-up after primary transanal pullthrough in TCA: (**a**) stooling; (**b**) perianal skin condition; (**c**) number of defecations.

We see children according to our standard follow-up schedule and offer telemedicine support when needed. In our cohort, we report data with a median follow of 20 months (1–28 months) after pullthrough and a median age of 45 months (11–142). We initially observe a mean of six defecations/day, which gradually decreases. Initially, a greater occurence of perianal rash is reported, and at 15 months follow-up, the majority has normal perianal skin, see Figure 2. 

## 4. Discussion

Our series of 17 new patients with total colonic aganglionosis within the last 2 years contributes to the understanding of this rare disease. All patients were born in external hospitals and received their initial care locally. Since complex colorectal diseases are not yet formally centralized in Germany [15], most patients were self-referred at a median age of 10.75 months.

### 4.1. Patient Characteristics

In our patient cohort, we found a 2.6:1 male-to-female sex ratio with 5 females and 13 males. In total colonic aganglionosis, a male/female distribution of 2:1 has been described [2,8]. We can confirm the known association with genetic syndromes such as Shah-Waardenburg with two cases in our cohort; we also note that Trisomy 21 is less frequent in TCA than in standard Hirschsprung Disease [10]. Only one patient in our study has Trisomy 21.

Patients in our cohort had a median of 4 (2–7) prior surgeries with various complications, similar to the series described by Bischoff in 2011 [8]. Recognizing and diagnosing total colonic aganglionosis remains a challenge when noting the plethora of procedures children undergo before the correct diagnosis is made [3,8,9]. Unfortunately, the rate of unnecessary surgery remains as high as described by Tsuji 25 years ago [7] and Bischoff 13 years ago [8]. In none of the children in this cohort was the diagnosis of TCA considered during the initial surgery. We find similar results for inadequate biopsies at prior surgeries in 15/18 cases, 3/18 stoma closures after neonatal acute abdomen without a diagnosis, 8/18 stoma placements in aganglionic parts of the bowel, 2/18 stoma placements too proximal, and 10/18 general stoma problems such as prolapse or stenosis. These inappropriate surgeries cause a variety of unnecessary surgeries and complications in children with total colonic aganglionosis. 

Analyzing our patient cohort operated with the same standard approach within two years allows us to evaluate our pathway of care for TCA. We include pre-pullthrough management, perioperative management, and postoperative follow-up for primary pullthrough.

### 4.2. Pre-Pullthrough Management and Primary Pullthrough

The remaining small bowel should in most cases allow the child to gradually achieve enteral autonomy. The level of normal ganglionic bowel should be established soon after diagnosis to plan the prehabilitation before pullthrough surgery [4]. Our cohort had a mean of 200 cm of ganglionic bowel available, assessed with direct measurement. Some children may require transient parental nutritional support in the early neonatal and infant period, which should be weaned at the time of pullthrough surgery. Other groups report the transient use of PN even after pullthrough [3,4,11]. A functioning stoma will eventually allow the child to thrive. Theoretically, once the child has a working ileostomy and is fully enteral-fed, this is a medically acceptable condition. It is important to understand that ileoanal pullthrough does not change the length of the bowel nor improve absorption or stool consistency. Ileoanal pullthrough simply changes the position of the ileum from the abdominal wall to the anal canal. Some diaper rash is expected, and families need to be prepared for this temporary rash [8,12].

In our cohort, we perform the primary pullthrough once the patients and families are ready at a median age of 16.75 months. Previously, the ideal age was considered above 3 years when the child had reached toilet training for urine, but we agree with those who have shifted to lower ages. Once the child is fully enteral-fed, and thriving, the stool has become creamy, and the family is ready for reconstruction and aware of the inevitable temporary perianal rash, the patient is suitable for ileoanal pullthrough [12]. We believe that the time when you think you do not want to change anything because everything is fine is the right time to perform the ileoanal pullthrough procedure. 

Training to tolerate the use of a transanal catheter (“catheter training”) is mandatory to adequately prepare for postoperative transanal irrigation, which is used for bowel management after pullthrough surgery [8,13]. We ask families to regularly introduce a soft silicone catheter at home before pullthrough surgery to gain acceptance of the child, similarly to the procedure for regular Hirschsprung Disease patients with a pre-existing stoma [13].

### 4.3. Key Element in Case of Mapping and Stoma Relocation

The initial surgeries and stoma formation are performed by teams elsewhere not seldom without a definitive diagnosis. Stoma mislocation occurs frequently and is observed in 10/18 (55.5%) cases in our series.

If the stoma is too proximal, the absorptive capacity is missed and can be recruited by placing the stoma more distally with the immediate control of intraoperative biopsies. On the other hand, a stoma in the functional obstructed aganglionic segment evokes recurrent episodes of obstruction with enterocolitis and ultimately affects thriving as well. We often observe external biopsy sites with non-absorbable sutures for marking the reasons causing remarkable adhesions, and we only perform biopsies with absorbable inverted sutures. Even the perforation of loop sutures has been reported. We urge care with biopsies during the mapping process [8]. During the mapping procedure, the existing small bowel length should be carefully measured and documented. 

### 4.4. Reconstructive Surgery Primary Pullthrough

The key element of reconstructive surgery is the removal of the aganglionic colon and the aganglionic small bowel. The unused aganglionic colon is usually small in caliber and contains inspissated mucus. We observe a high degree of atrophy in older children with longstanding fecal diversion. However, in the group of primary pullthrough, we do not observe any problems from the anastomosis side, although it is difficult to draw statistical conclusions from this case seria.

**Figure 3:**
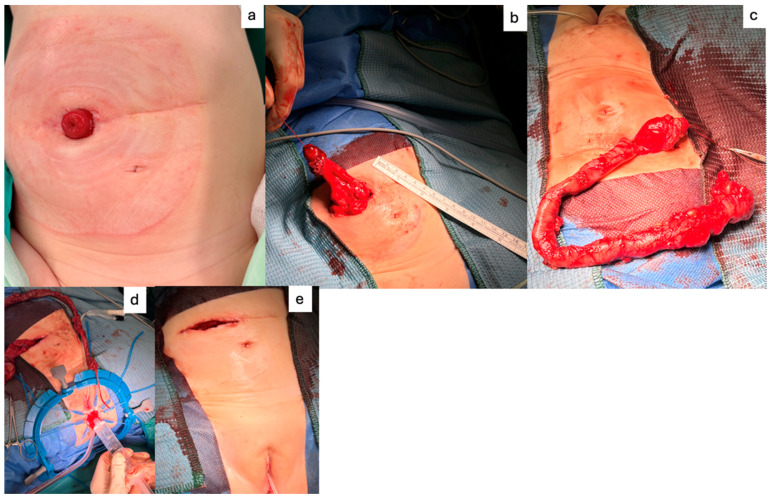
Stoma side incision access and ileoanal pullthrough: (**a**) ostomy, (**b**) stoma mobilization; (**c**) colectomy via stoma side; (**d**) transanal dissection; (**e**) before skin closure.

In general, we approach reconstructive surgery with the colectomy via the existing stoma wound providing access hence avoiding a large incision. We aim to limit the operative time by working with two teams simultaneously. Our data support that total colectomy with straight ileoanal pullthrough should be the treatment of choice as it is easier to perform and has fewer short- and long-term complications than pouch creation [8,12,16]. The ostomy will become the pullthrough bowel (see Figure 3).

We aim to avoid a covering stoma to allow the pullthrough to immediate function and, with this stool passage, also to prevent any anastomosis stricture formation. Subsequently, no anal dilatations are needed. The advantage of moving to younger ages is to minimize the time of the defunctioned unused distal bowel causing atrophy at the very distal rectal mucosa. This approach has worked in all cases of primary pullthrough in our cohort. However, we realize that especially in redo cases with a long-standing diverted stoma, the distal anorectum is very friable. The risk of wound dehiscence at the distal ileoanal anastomosis for redo cases without a temporary diverting stoma is up to 50% in the early cohort. Therefore, we switched to use a temporary diversion stoma (4–8 weeks), which not only protects the wound healing but also allows for a faster recovery from redo surgery.

Particularly in smaller children, the entire abdominal cavity can be reached via the ostomy side. When the patient has reached a certain size, we sometimes use another pre-existing scar to complete the access, usually to the pelvis. Teamwork with the anesthesia team is crucial to achieving the needed deep anesthesia that allows access to the entire abdominal cavity. The transanal pullthrough is prepared sequentially operating in two teams. One team conducts the transanal preparation while the other team performs the colectomy. We acknowledge that other teams may use laparoscopy for the colectomy; however, as our operative strategy includes removing the stoma as well, we use the peristomal incision to gain access. The preservation of the dentate line and sphincters is key to continence, and an anal retractor (Lone Star Retractor System™: CooperSurgical Inc., Trumbull, CT, USA) is used to protect the distal anal canal. The anastomosis is placed approximately 3 cm above the dentate line as opposed to 1 cm above in standard Hirschsprung cases. There is no advantage in leaving a longer aganglionic segment, which only causes obstruction and has to be removed later during revision [10,12,17].

### 4.5. Immediate Post-Pullthrough Management

#### 4.5.1. Mitigating Operative Stress

The physiologic catabolic response to surgery may be exacerbated by perioperative pain, so adequate pain management is extremely important. The operation usually involves complete adhesiolysis and repositioning of the small bowel in its new position. Opioid-based analgesia according to the WHO pain ladder combined with perioperative caudal anesthesia should mitigate pain.

#### 4.5.2. Immediate Bowel Management

Rehabilitation is a well-accepted concept to facilitate timely recovery after surgery. In total colonic aganglionosis, the pulled-through small bowel must learn to function as a distal bowel. Abdominal distension, mainly due to retained air, causes discomfort, and the rapid restoration of adequate peristalsis will help during the recovery period.

Subsequently, we note a clear benefit of rectal irrigations mainly to allow the decompression of air from the first postoperative day, likewise in standard Hirschsprung Disease [13]. The first postoperative irrigation is performed by the surgeons themselves, as the child is generally accustomed to and comfortable with transanal irrigation.

We keep the children on perioperative antibiotic prophylaxes for a minimum of 3 days and depending on the inflammatory response. We note a median duration of 4.5 days in our seria.

#### 4.5.3. Early Post-Discharge Management

The focus of early postoperative follow-up is on (a) stool frequency, (b) perianal excoriation, and (c) enterocolitis.

Like the groups of Levitt or Bischoff, we continue with transanal irrigation [12], initially using the passive method with a silicone Foley catheter and a syringe, and may switch to active transanal irrigation (e.g., with Peristeen^®^: Coloplast, Hamburg, Germany) during follow-up. The most troublesome condition is the perianal rash, which eventually gets better with time [12], often caused by many small-volume, inefficient stools. Irrigation helps to reduce the frequency of stools, and some children benefit from slowing motility with Loperamide [12]. Probiotics are used in 30% of patients with Hirschsprung [13], and in our daily practice, we see some benefit from probiotics, but the overall evidence remains uncertain, and further detailed research would be needed [18]. 

### 4.6. Follow-Up

Our institutional follow-up program includes on-site and telemedicine follow-up with easy access for families to the medical team in case of concerns.

### 4.7. Recommendations for Clinical Practice from Rostock TCA Team

**Figure 4:**
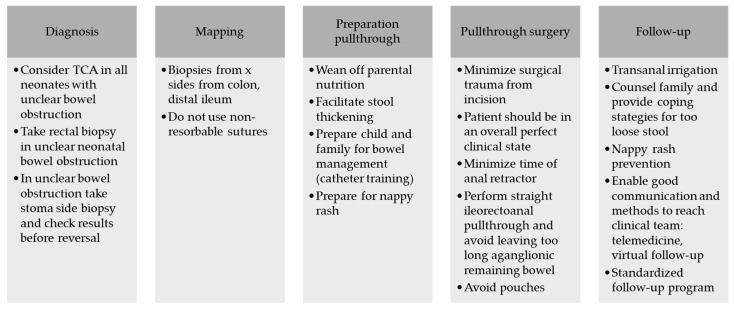
Recommendations for clinical practice from Rostock TCA team.

We propose to consider the following items to ensure a adaeqaute preparation and surgical strategy can be achieved, see Figure 4.

### 4.8. Limitations and Strengths

We recognize that patients who come to us with total colonic diagnosis and initial treatment elsewhere represent a highly skewed group that is not necessarily representative. Unfortunately, none of the children were initially seen by us in the immediate neonatal period. As with all reviews of clinical data, our data are only as good as the records. Our cohort is a large cohort undergoing pullthrough surgery in a short period. This excludes the fact that overall medical advances are changing.

## 5. Conclusions

Recognizing the rare and complex nature of total colonic aganglionosis, multidisciplinary holistic management, prehabilitation, and follow-up by a dedicated team is essential to provide the best quality of life. Providing a functional stoma that allows the child to thrive and gain enteral autonomy paves the way for a successful ileoanal pullthrough. Establishing the knowledge and tolerance of transanal irrigation helps to ensure postoperative bowel management. A straight ileoanal pullthrough without a protective stoma should be attempted in primary cases.

High stool frequency, loose stools, and perianal rash are associated with the nature of ileoanal pullthrough. The severity can be reduced by bowel monitoring, nutritional advice, and close follow-up by a dedicated specialist team. These unavoidable side effects will improve with time.

## Figures and Tables

**Table 1:** Bowel management protocol for TCA at the Colorectal Centre Rostock: preparation for primary pullthrough for TCA.

	Stoma Present
Perianal skin protection	Transfer stoma effluent to perineal skin
Perianal hygiene	Rinse, no whipping Happy Po^®^
Dietary advice	Stool thickening: early carrot, psyllium husk Increase salt intake Potentially oral NaCl supplementation if urine sodium below (20 mmol/L) If older, low-fiber diet
High stoma output	Replacement with oral rehydration solution, consider antibiotics for dysbiosis
Acquisition of tolerance for catheter	Insert rectal tube intermittently
Microbiome	Probiotics if not breastfeeding
Antibiotics	1st line: Metronidazole p.o./in stoma 2nd line: Amoxiclav^®^ p.o.

**Table 2:** Enhanced recovery in primary pullthrough for total colonic aganglionosis.

Analgesia	Postoperative Nutrition	Postoperative Mobilization	Applicability of Minimally Invasive Surgery	Limit Indwelling Plastic
Caudal block initially including opioids	Start clear fluids in recovery Allow small amounts of milk and solid food after surgery	Allow immediate patient-driven mobilization Start to mobilize from day 1	Limit time of Lone Star^®^ retractor Aim for quick procedure time, e.g., by working with two teams Limited incision at stoma site	No nasogastric tube No drains Urinary catheter time limited (until patient weaned off opioids)

**Table 3:** Follow-up protocol for total colonic aganglionosis (at the Colorectal Center Rostock) after primary pullthrough for TCA.

	All	Too Many Stools	Perianal skin Excoriation	Enterocolitis
Perianal skin protection	Barrier crème	(a) Barrier crème (b) Increase TAI	(a) Barrier crème (b) Increase TAI	
Perianal hygiene	Showering after defecation, using water to rinse, e.g., HappyPo © No wiping	Increase Transanal Irrigation to max 2 times per day	No wiping	
Dietary advice	Low-fiber diet	Stool thickening, Early carrot	Alkaline diet, e.g., dry chickpeas Early carrot	
Watery diarrhea	Oral rehydration solution, consider HAEC			
Transanal irrigation (TAI)	Once daily	Increase irrigation to 2 times per day	Increase irrigation to 2 times daily	Increase irrigation to 2 times daily
Microbiome	Probiotics			
Antibiotics for HAEC				1st line: Metronidazole p.o. 2nd line: Amoxiclav^®^ p.o.
Loperamide		Only if increased TAI is not effectively reducing frequency	Only if increased TAI is not effectively reducing frequency	
Protone Pump Inhibitor		Considered when TAI is not showing sufficient results	Considered when TAI is not showing sufficient results	

**Table 4:** Patient characteristics at presentation.

	All N = 18
Gender	5 female 13 male
Family History for HD Parent (multiple answers possible) Sibling (multiple answers possible)	6/18 2/18 6/18
Genetic comorbidity Trisomy 21 Shah-Waardenburg	1 3 2
Prior abdominal surgeries (median, SD)	4 (2–7)
Age referral Months Median	10.75 (2–88)
Nutrition at referral Enteral Partial parenteral Full parenteral	12 5 1
Toilet-trained for urine at presentation	

**Table 5:** Patient findings during mapping.

	Mapping N = 12
Mean age at mapping (months)	11 (3–54)
Mean body weight at mapping (kg)	11.2 (7–15)
Mean hemoglobin (mmol/l)	7 (6.8–8.1)
Mean length of aganglionic ileum (cm)	15 (5–93)
Extension TCA Only colon Colon and up to 20 cm Ileum Colona and 21–50 cm Ileum Colon and more than 51 cm Ileum	1 9 3 2
Mean length of remaining ganglionic bowel (cm) for TCA	178 (110–254)
Stoma relocated during Mapping More proximal More distal Same location but local stoma problem	10/12 83% 5/10 3/10
Stoma problems (multiple possible) Stenose Prolapse Obstruction	2 2 7
Complication mapping (Clavien–Madadi classification)	none

**Table 6:** Patient characteristics at pullthrough surgery.

	Primary Pullthrough N = 11
Median age at primary pullthrough (month)	18 (10–133)
Age at pullthrough (years) <1 1–3 >4	3 4 4
Mean body weight pullthrough (kg)	14.8 (9–29)
Gender m:f	7:4
Toilet-trained for urine at time of pullthrough	4/11
Current nutrition situation Fully enteral Partial parental nutrition	11 0
Prior mapping by our Team	7/10, 70%
Mean length of aganglionic ileum (cm)	16 (5–60)
Median length of ganglionic ileum Treitz to Ileostomy median (cm)	226 (140–254)
Colectomy completed	11/11
Mean length of aganglionic colon (cm)	43 (24–64)
Hemoglobin pullthrough mean (mmol/L)	6.9 (5.4–8.5)
Perioperative transfusion needed	6/11, 55%
of which 1 transfusion used	5/6
of which 2 transfusions used	1/6
Mean length of perioperative antibiotic use (days)	5.7d (3–10 d)
Ileoanal pullthrough stoma side	11
Covering stoma at time of pullthrough	0
Operative time	3:15 (2:25–4:17)
Complication mapping (Clavien–Madadi classification) I II III IV	1 3: conversion antibiotic 0 0

## Data Availability

The data presented in this study are available on request from the corresponding author. The data is recorded in our Rostock Colorectal Hirschsprung patient registry due to privacy or ethical restrictions.

## Abbreviations

ICV	Ileocecal valve
PN	Parenteral nutrition
TAI	Transanal irrigation
TCA	Total colonic aganglionosis
